# A Contact-Mode Triboelectric Nanogenerator for Energy Harvesting from Marine Pipe Vibrations

**DOI:** 10.3390/s21041514

**Published:** 2021-02-22

**Authors:** Rui Li, He Zhang, Li Wang, Guohua Liu

**Affiliations:** College of Civil Engineering & Architecture, Zhejiang University, 866 Yuhangtang Road, Hangzhou 310058, China; 21812222@zju.edu.cn (R.L.); 11812069@zju.edu.cn (L.W.); zjuliugh@zju.edu.cn (G.L.)

**Keywords:** mechanical energy harvesting, triboelectric nanogenerator, marine pipelines, optimization design, scaling law

## Abstract

Structural health monitoring is of great significance to ensure the safety of marine pipes, while powering the required monitoring sensors remains a problem because the ocean environment is not amenable to the traditional ways of providing an external power supply. However, mechanical energy due to the vortex-induced vibration of pipelines may be harvested to power those sensors, which is a convenient, economic and environmentally friendly way. We here exploit a contact-separation mode triboelectric nanogenerator (TENG) to create an efficient energy harvester to transform the mechanical energy of vibrating pipes into electrical energy. The TENG device is composed of a tribo-pair of dielectric material films that is connected to a mass-spring base to guarantee the contact-separation motions of the tribo-pair. Experimental tests are conducted to demonstrate the output performance and long-term durability of the TENG device by attaching it to a sample pipe. A theoretical model for the energy harvesting system is developed for predicting the electrical output performance of the device. It is established that the normalized output power depends only on two compound variables with all typical factors taken into consideration simultaneously. The simple scale law is useful to reveal the underlying mechanism of the device and can guideline the optimization of the device based on multi-parameters analyses. The results here may provide references for designing contact-mode TENG energy harvesting devices based on the vibration of marine pipes and similar structures.

## 1. Introduction

Vortex-induced vibration (VIV) [1] of marine pipes is a typical type of vibration caused by the vortex shedding phenomenon of the fluid flowing through the pipe. VIV may finally lead to fatigue or damage of the pipe after long-term service in the deep ocean environment. To ensure the safety of marine pipes, health monitoring systems are of great importance. Distributed optical fiber sensors [2,3,4,5] are the most popular sensing devices for pipeline status monitoring. However, these types of devices are usually driven by traditional external power supplies. Considering the high cost on infrastructure for traditional power supplies and the corresponding energy loss in the process of long-distance power transmission, it is essential to develop self-powered sensing technologies [6] to free the health monitoring system from traditional power supplies.

A self-powered system may operate sustainably by scavenging electrical energy from the ambient environment. The technologies of piezoelectric generators [7,8,9,10] and triboelectric generators (TENG) [11,12,13] represent the most popular technologies for harvesting energy from environmental mechanical motions. Piezoelectric generators have been proposed as a good energy harvesting strategy using the vibrations of pipelines [14,15,16,17] with a power conversion efficiency of more than 50% [18]. In contrast to the piezoelectric effect, the TENG technology, pioneered by Wang and his co-workers [19], is more feasible for scavenging energy from environment vibrations with low frequencies, such as ocean waves and the vibrations of pipelines, bridges, and the like. As a kind of blue energy, the ocean wave energy exhibits a series of merits like sustainability in application and friendliness for the environment. Recently, TENG devices with different designs were developed in this application area [20,21,22,23,24,25,26,27,28]. Chen et al. [20] proposed a network design made of TENGs for large-scale harvesting of kinetic water energy. Subsequently, Jiang et al. [29] improved the energy conversion efficiency of TENG devices by developing a wavy-structured conductor-dielectric-conductor film. To further enhance the output performance, Zhao et al. [30] modified the surfaces of tribo-layers by introducing a nanostructured morphology. To avoid the influence of high humidity, Xu et al. [31] developed a fully packaged TENG. The energy harvested by each TENG from ocean waves is roughly capable of powering a sensor. To achieve more electric power, a network of TENG devices floating on the water surface was proposed [20]. The output power of this device combination can reach 1.15 MW and the instantaneous energy conversion efficiency reaches 55% for a one-kilometer square water surface.

In addition to ocean waves, vibrating marine pipes are also an effective host for energy harvesting. For the purpose of providing a power source for pipeline monitoring systems, we propose to exploit contact-mode TENG to create a high-performance energy harvester. This device can harvest the mechanical energy of vibrating pipes by converting it into electric power, which may offer then a power supply for the pipeline health monitoring system. The device is supposed to be attached on the surface of the pipeline, so that it may vibrate with the pipeline due to the ocean flow. It is designed with two dielectric films, which are connected by a mass-spring system for the restoring force. An alternating current will be produced by the device during the contact-separation oscillation process [32] induced by the pipe vibrations. A series of experimental tests will be carried out to investigate the output performance of the TENG device due to different pipe motions and electrical circuit resistance. To validate the feasibility of the TENG device, a theoretical model is established to predict dynamical responses of pipes, the oscillation of the mass-spring base and the electric output of TENG devices. Parameter analyses are performed to examine the influences of the installation position of the device on the pipe, the natural frequency of the mass-spring base, the effective thickness of the dielectric films and the circuit loading resistances. Finally, a simple scaling law is established based on the multi-parameter analyses to reveal the underlying correlation between the normalized output power and normalized system parameters. The results indicate that the application of TENG has potential in propelling wireless sensing and battery free detection in marine pipeline monitoring systems.

## 2. Materials and Methods

**Figure 1:**
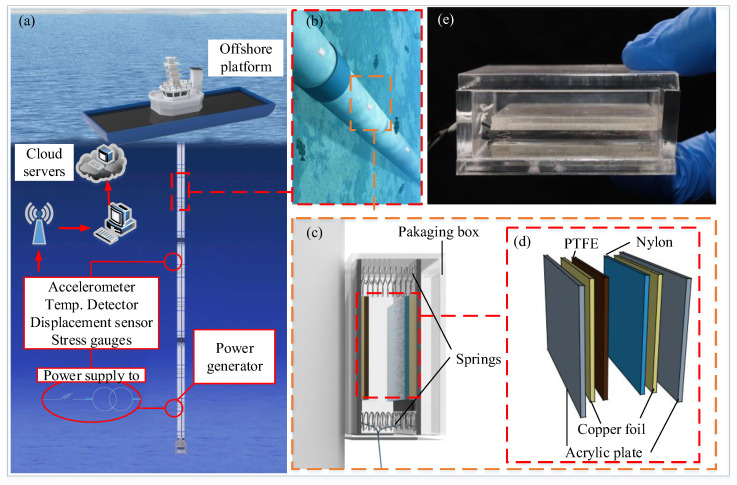
A sketch of the developed TENG devices in application of ocean pipe vibration energy harvesting. (**a**,**b**) are the perspective of the device arrangement at pipe; (**c**,**d**) are illustration of the energy harvesting system and detailed structural diagram of the tribo-pair of TENG device; (**e**) is the fabrication of the TENG device sealed by a waterproof box.

TENG devices are used to scavenge mechanical energy of a vibrating pipeline which can power the monitoring system to realize the sensing and signal transmission. The TENG device consists of a tribo-pair, two electrodes, two acrylic plates, several springs and a waterproof package (Figure 1). The movable acrylic plate and four springs form a mass-spring base, which is designed as the carrier for the tribo-pair to facilitate the energy conversion through the contact-separation oscillations. The acrylic plates serve as the carrier of the tribo-pair, which make it become flat so that the tribo-pair may contact intimately and prevent the leakage of electrons from copper foil to the external environment. The copper foils are bonded outside of the tribo-pair and inside of the acrylic plates as the conductive electrodes. Here we use polytetrafluoroethylene (PTFE) and nylon as the dielectric materials of the tribo-pair. The springs fixed between the two acrylic plates offer the restoring force when the tribo-pair are forced into contact. The whole TENG device is sealed by a waterproof box to protect the TENG device and restrict the lateral deformation of TENG devices.

**Figure 2:**
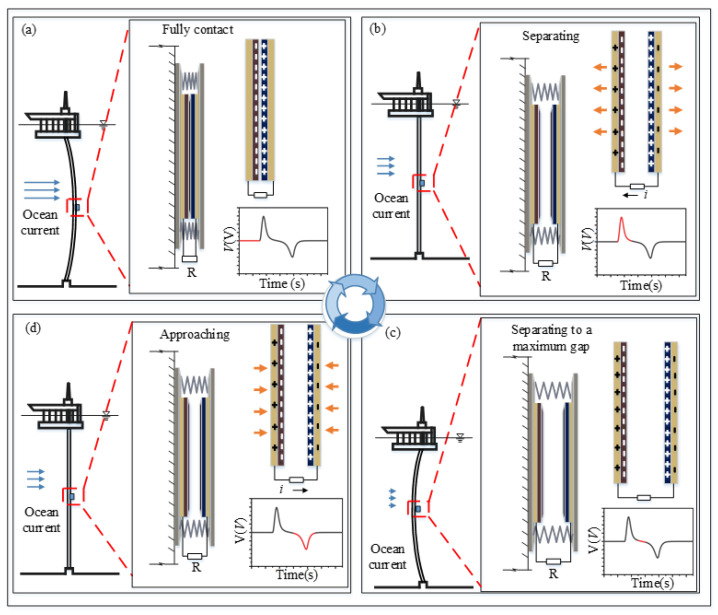
Four process steps for a TENG device to convert the mechanical energy of a vibrating pipe into electricity. (**a**) In the fully contact process, the pipe is bended under the current excitation, and the tribo-pair are getting into contact; (**b**) In the separating process, the current excitation is reduced, and the pipe reverts back and the tribo-pair starts to separate; (**c**) In the separating to a maximal gap process, the current excitation keeps reducing, and the pipe keeps bending in the opposite direction due to inertia and the tribo-pair gets separated to the maximum distance; (**d**) In the approaching process, the current excitation increases, and the pipe returns to its original state and the dielectric material films of the tribo-pair begins to approach.

When TENG devices are fixed on the outer surface of the pipeline, the vibration of the host pipe will force the TENG devices into periodic oscillations. In each working cycle, four stages occur in sequence: fully contacting, separating, separating to a maximal gap, approaching. In the first stage (Figure 2a), the pipe is bent under the ocean current excitation and the tribo-pair comes into contact. The electrons transfer from nylon to PTFE due to static induction, so that the two layers carry equivalent opposite charges. In stage II (Figure 2b), the current excitation is reduced, and the pipe reverts back and the tribo-pair begins to separate. A potential drop will be generated between electrodes deposited on the top and the bottom surfaces of two dielectric films. To balance the potential drop, free electrons in the electrode attached to PTFE will flow to the electrode attached to nylon through external circuit, which generates a voltage pulse with a positive amplitude. In stage III (Figure 2c), the current excitation keeps reducing, and the pipe keeps bending in the opposite direction due to inertia and the tribo-pair gets separated to the maximum distance. The free electrons stop transferring and the tribo-pair reaches a new electrical equilibrium. In stage IV (Figure 2d), with the increasing of the ocean current excitation, the pipe bends in the flow direction and the dielectric material films of the tribo-pair begins to approach. Free electrons come back to the electrode connected to PTFE when the tribo-pair contact again (Figure 2a), and a negative voltage pulse generates. In this way, an alternating output voltage in external circuit of TENG devices occurs due to the flow- induced vibrations of the pipe.

## 3. Experimental Test

**Figure 3:**
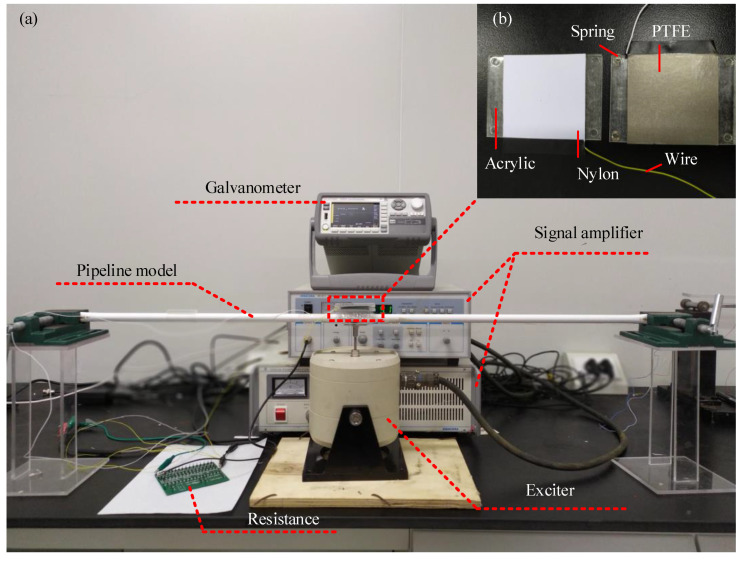
A photo of the experimental setup. (**a**) The experimental setup consists of a PVC pipe, two bearings, an exciter, a galvanometer, a TENG device and external resistances; (**b**) The PTFE layer, nylon layer and the acrylic base.

To investigate the output performance of a TENG device, a series of experimental tests will be carried out to test the electrical output of the TENG device due to different pipe motions and electrical circuit resistances. The experimental setup includes the TENG device, a polyvinyl chloride (PVC) pipe, a vibration exciter, a signal amplifier and a galvanometer and an oscilloscope (Figure 3). The simply supported PVC pipe, of which the two ends are hinged by a pair of pliers and the middle is adhered to the exciter, is used as the host for TENG devices. The pipe is 1.2 m in length and 0.01 m in diameter. The TENG device is pasted on the top of the pipe. The smooth nylon film with a thickness of 100 μm/smooth PTFE film with a thickness of 50 μm (triboelectric layers) are utilized for the tribo-pair. In the test, a periodic harmonic load with the loading amplitude of 3 mm and frequency of 9 Hz is applied on the middle of pipe by the exciter. Other corresponding parameters are presented in Table 1. To demonstrate the practical use of the proposed TENG device, the TENG attached on the simply supported PVC pipe is loaded to light up 14 LEDs with the external resistance of 1.14 MΩ, as shown in Video S1.

**Figure 4:**
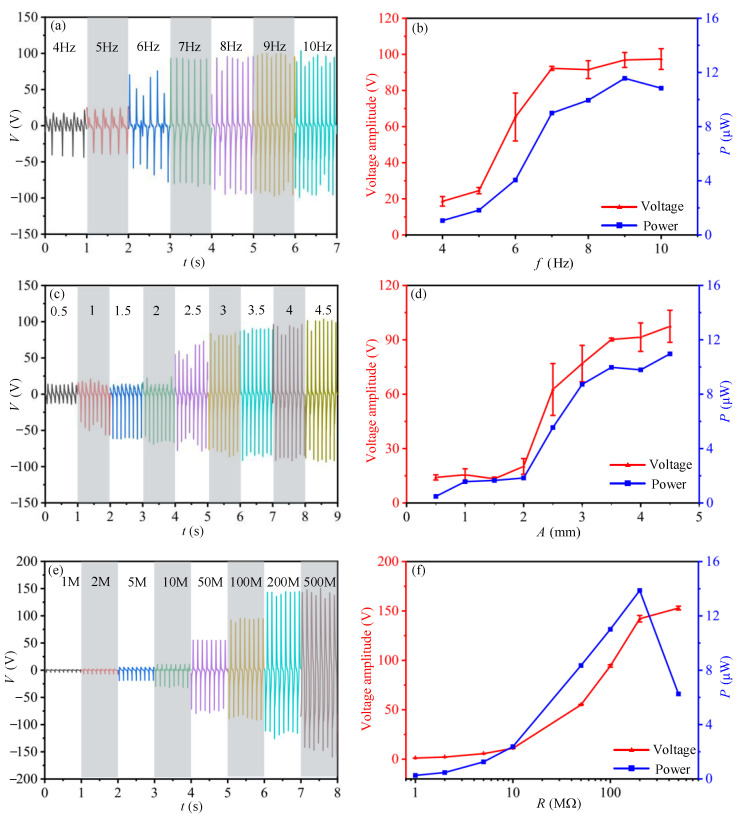
Electric output of TENG device at different physical and electrical conditions. (**a**,**b**) are the influence of mechanical loading frequency ranging from 4 Hz–10 Hz on the output performance of TENG device; (**c**,**d**) are the influence of mechanical loading amplitude applied on the pipe ranging from 0.5 mm–4.5 mm on the output performance of TENG device; (**e**,**f**) are the influence of circuit loading resistance ranging from 1 MΩ–500 MΩ on the output performance of the TENG device.

As the TENG device is attached at the pipeline, the vibration amplitude and frequency of pipeline are considered crucial factors affecting the output performance of TENG device. To investigate the relationship between them, the output current of TENG device attached at the pipe is tested under different mechanical loads. Firstly, the output current of the TENG devices under different excitation frequencies is measured and the output voltage is obtained according to Ohm’s law: *V = IR*. The external excitation starts with the loading frequency varying from 4 Hz to 10 Hz under a loading amplitude of 4 mm. The results (Figure 4a,b) show that the voltage amplitude increases with loading frequency in the range of 4 Hz to 7 Hz, and then reaches a relatively stable value (about 102 V) when the frequency exceeds 7 Hz. The average output power of TENG is calculated according to the output voltage signals. As Figure 4b shows, the maximum average output power (11.6 μW) occurs when the excitation frequency is 9 Hz.

Next, the output performance of TENG device due to different pipe vibration amplitudes is tested. The exciter is set up to make the pipe vibrate with an amplitude in the range of 0.5–4.5 mm and a frequency of 8 Hz. The results (Figure 4c,d) show that the voltage amplitude increases with the vibration amplitude of pipe. The maximum average output power (11.2 μW) occurs when the vibration amplitude of the pipe is 4.5 mm.

To further study the output performance of the TENG device due to different electrical load resistances, the output current of TENG device is measured as the external loading resistors range from 1 MΩ to 500 MΩ. According to the two tests above (Figure 4a–d), a mechanical excitation with frequency of 8 Hz and amplitude of 4 mm is facilitated by the exciter (Figure 4e,f). The results show that the peak voltage increases as the load resistance increases, and reaches a stable value at load resistance of 200 MΩ. The average output power increases firstly and then decreases with the peak of 14.0 μW at a circuit resistance of 200 MΩ.

**Figure 5:**
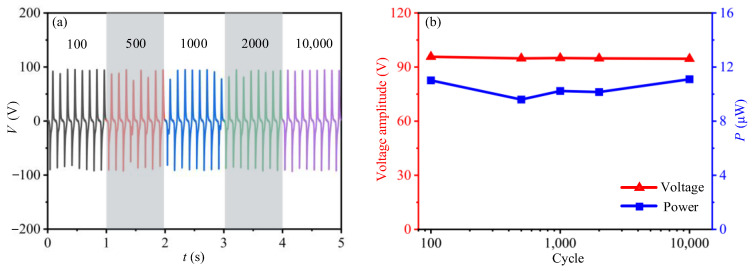
Output performance of TENG device after a certain period of operation. (**a**) is the time history results of the output current of TENG device after certain cycles of operation; (**b**) is influence of the loading cycles on the peak output current and average power of TENG device.

The long-term durability of the TENG device is also a critical issue for a mechanical energy harvester. Thus, the tests for measuring the cycling performance of the TENG device during 10,000 cycles are carried out at the loading frequency of 8 Hz and excitation amplitude of 3.5 mm (Figure 5a,b). Figure 5a presents the output current signals of TENG device after 100, 500, 1000, 2000 and 10,000 cycles, respectively. In Figure 5b, both the peak current and the average output power has no significant degradation after 10,000 reciprocating cycles, indicating an excellent stability and durability of the TENG device.

In practice, the devices that need to be powered in the wireless pipeline monitoring system mainly include sensors and wireless signal transmission devices, for which a microwatt level power consumption can be estimated [33]. According to the measured output of the TENG fabricated in this paper, the output may meet the power consumption requirements of these devices. In addition, the TENGs could be electrically connected in parallel or in series to form a network on the surface of a pipeline. The network will greatly enhance the output of the energy harvesting system so that it could provide an adequate green energy supply for the monitoring system instead of using a traditional power supply.

## 4. Theoretical Modelling

To understand the operation mechanism of TENG in scavenging the mechanical energy from marine pipes and provide a better optimization method for the structure, material and electric circuit of the energy harvesting system, we establish a theoretical model of the energy harvesting system and predict the electrical output of the TENG device theoretically.

### 4.1. Dynamic Responses of the Pipe and the Mass-Spring Base

**Figure 6:**
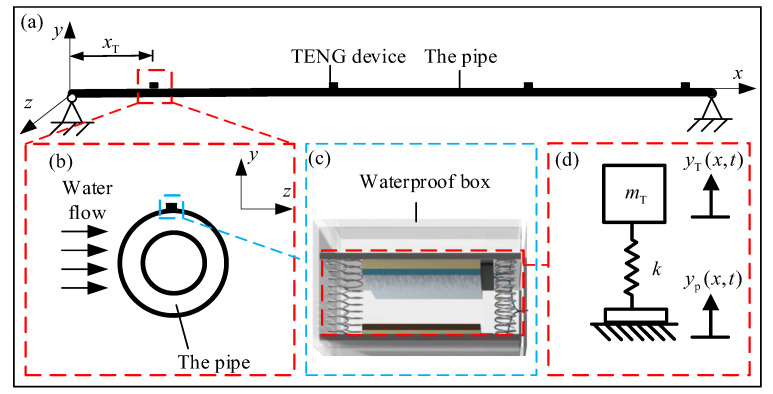
Schematic design of the pipe host. (**a**) the integral pipe which is assumed to be a simply supported beam; (**b**) the cross section of the pipe; (**c**) TENG device sealed by a water-proof box; (**d**) the schematic design of mass-spring base of TENG device.

The schematic structural illustration of the energy harvesting system is shown in Figure 6. Firstly, the pipe is modelled as a simply supported Bernoulli-Euler beam (Figure 6a) with several TENG devices attached on it. A Cartesian coordinate system is used with the origin at the left end of the beam with the axial direction of the beam the *x*-axis. The incoming flow direction is assumed in the direction of *z*-axis, with the lateral response of the pipe in *y* direction calculated. According to the Euler-Bernoulli beam theory [34], the governing equation of the simply-supported cantilever beam can be written as Equation (1). The vibration equations for TENG device is written as Equation (2) based on D’Alembert’s principle:

(1) $$\frac{\partial^{2}}{\partial x^{2}}[EI\frac{\partial^{2}y_{p}(x,t)}{\partial x^{2}}]+\bar{m}\frac{\partial^{2}y_{p}(x,t)}{\partial t^{2}}+c_{p}\frac{\partial y_{p}(x,t)}{\partial t}=p(t)$$

(2) $$m_{T}{\ddot{y}}_{T}(t)+ky_{T}(t)=-m_{T}{\ddot{y}}_{p}(x_{T},t)$$

in which *E* is the elastic modulus, *I* the cross-sectional moment of inertia, $\bar{m}$ the linear mass density, $c_{p}$ the damping coefficient, $y_{p}(x,t)$ the cross-line displacement vector of pipe, and *t* represents the derivation of time, p(t) the hydrodynamic fluid forces acted on pipe, $y_{T}(t)$ the displacement vector of TENG device, $m_{T}$ the mass of the TENG base, $x_{T}$ the installation position of the TENG devices on the pipe, and k the springs’ stiffness.

According to the wake oscillator model [35], the hydrodynamic fluid forces on pipeline may be expressed as:

(3) $$p(t)=\frac{1}{2}C_{L}\rho_{w}DU^{2}\cos2\pi f_{s}t$$

in which $C_{L}$ is the lift coefficient, $\rho_{w}$ the water density, *D* the diameter of the pipe, *U* the flow velocity, $f_{s}=S_{t}\cdot U/D$ the Strouhal frequency, and $S_{t}$ the non-dimensional Strouhal number ($S_{t}=0.2$).

Based on the mode superposition method, a generalized expression for the displacement of the pipe is written as:

(4) $$y_{p}(x,t)=\sum_{j=1}^{n}\phi_{j}(x)q_{j}(t)$$

in which, $\phi_{j}(x)$ is the *j*-th mode shape, $q_{j}(t)$ the generalized displacement of *j*-th normal mode in time domain. For simply supported beam, $\phi_{j}(x)=\sin\frac{j\pi }{L}x$, in which, *L* is the length of the pipe.

Substituting Equations (3) and (4) into Equation (2), we have:

(5) $${\ddot{q}}_{j}(t)+2\zeta_{pj}\lambda_{pj}{\dot{q}}_{j}+\lambda_{pj}^{2}q_{j}(t)=P_{j}(t)/M_{j}$$

in which, $\lambda_{pj}={\left(j\pi \right)}^{2}\sqrt{\frac{EI}{\bar{m}L^{4}}}$ is the natural circular frequency of pipe, $\zeta_{pj}$ is the hysteretic damping coefficient of the pipe defined by $\zeta_{pj}=\frac{1}{2}\left(0.008j^{2}+\frac{0.032}{j^{2}}\right)$ [33], $P_{j}(t)=\int_{0}^{L}p(t)\phi_{j}(x)dx=\frac{LC_{L}\rho_{w}DU^{2}\left(1-{\left(-1\right)}^{j}\right)}{2j\pi }\sin\left(2\pi S_{t}\frac{U}{D}t+\frac{\pi }{2}\right)$ and $M_{j}=\int_{0}^{L}\bar{m}\phi_{j}^{2}(x)dx=\bar{m}\frac{L}{2}$ respectively the generalized loading and generalized mass of the *j*-th normal mode, *L* the length of the pipe.

By solving Equation (5), the generalized displacement of the *j*-th normal mode will be:

(6) $$q_{j}(t)=\frac{L^{4}C_{L}\rho_{w}DU^{2}(1-\cos j\pi )\sin\left(2\pi S_{t}\frac{U}{D}t-\beta_{j}\right)}{j^{5}\pi^{5}EI\sqrt{{\left(1-\gamma_{pj}^{2}\right)}^{2}+{\left(2\zeta_{pj}\gamma_{pj}\right)}^{2}}}$$

in which, $\beta_{j}=\arctan\frac{2\zeta_{pj}\gamma_{pj}}{1-\gamma_{pj}^{2}}$ is the phase difference of the generalized displacement of the *j*-th normal mode, $\gamma_{pj}=\frac{2\pi S_{t}U}{D\lambda_{pj}}$ the frequency ratio between the flow excitation and the *j*-th natural frequency of the pipe.

Substituting Equation (6) into Equation (4), the vibration displacement of the pipe will be:

(7) $$y_{p}\left(x,t\right)=\frac{L^{4}C_{L}\rho_{w}DU^{2}}{\pi^{5}EI}\sum_{j=1}^{n}\frac{\left(1-{\left(-1\right)}^{j}\right)\sin\left(2\pi S_{t}\frac{U}{D}t+\beta_{j}\right)\sin\frac{j\pi x}{L}}{j^{5}\sqrt{{\left(1-\gamma_{pj}^{2}\right)}^{2}+{\left(2\zeta_{pj}\gamma_{pj}\right)}^{2}}}$$

The dynamic oscillation of TENG is excited by the vibrations of the pipe, of which the displacement expression is shown in Equation (7). By introducing the vibration displacement of the pipe into the oscillation equation of TENG in Equation (2) and specifying the initial conditions, the vibration displacement of TENG will be obtained. Considering the initial condition that at t=0, two dielectric material films just get into contact, which can be expressed by $y_{T}(t=0)=0$. Substituting this initial condition and Equation (7) into Equation (2), the vibration displacement of the mass-spring base of TENG is:

(8) $$y_{T}(t)=\frac{L^{4}C_{L}\rho_{w}DU^{2}}{\pi^{5}EI\left(\frac{1}{\gamma_{T}^{2}}-1\right)}\sum_{j=1}^{n}\frac{\left(1-{\left(-1\right)}^{j}\right)\sin j\pi \frac{x_{T}}{L}\left(1+\sin\left(2\pi t+\beta_{j}\right)\right)}{j^{5}\sqrt{{\left(1-\gamma_{pj}^{2}\right)}^{2}+{\left(2\zeta_{pj}\gamma_{pj}\right)}^{2}}}$$

in which $\gamma_{T}$ is the frequency ratio between the flow excitation and the natural frequency of the mass-spring base.

### 4.2. The Electric Output of TENG Device

**Figure 7:**
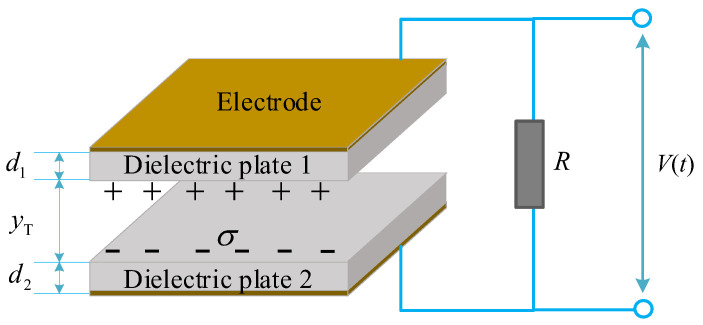
The schematic design of the tribo-pair of TENG device.

In Section 4.1, we have established the expressions of the oscillation displacement of the mass-spring base of TENG device, which denotes the variation of the surface gap between the two substrates in Figure 6c. As the dielectric plates and electrodes are closely attached at the substrates, the variation of the gap distance between the dielectric plates are equal to that of the two substrates ($y_{T}(t)$). The dielectric plates get into contact when $y_{T}(t)=0$, and get separated to the max gap when $y_{T}(t)$ reaches the maximum value. Substituting $y_{T}(t)$ into the basic *V-Q-x* relationship of TENG device [36,37,38] with a load resistance *R* (Figure 7), the output voltage of the tribo-pair (V(t)) is expressed as:

(9) $$\begin{array}{lll} V\left(t\right) & = & -\frac{\sigma d_{0}}{\varepsilon_{0}}+\frac{\sigma \left(d_{0}+y_{T}(t)\right)}{\varepsilon_{0}}\exp\left[-\frac{1}{RS\varepsilon_{0}}\left(d_{0}t+\int_{0}^{t}y_{T}(t)dz\right)\right]\\ & & +\frac{\sigma d_{0}}{\varepsilon_{0}}\frac{d_{0}+y_{T}(t)}{RS\varepsilon_{0}}\int_{0}^{t}\exp\left[\frac{1}{RS\varepsilon_{0}}\left(d_{0}\left(z-t\right)+\int_{t}^{z}y_{T}(t)dr\right)\right]dz \end{array}$$

here, *S* is the area size of the dielectric material film, $\varepsilon_{0}$ the dielectric constants of the air gap, σ the charge density of each dielectric material film, R the electrical circuit resistance, $d_{0}$ the effective thickness constant defined by $d_{0}=\frac{d_{1}}{\varepsilon_{r1}}+\frac{d_{2}}{\varepsilon_{r2}}$, $d_{1}$ and $d_{2}$ the thickness of two dielectric plates, $\varepsilon_{r1}$ and $\varepsilon_{r2}$ the relative dielectric constants.

The expression for average power of TENG with an external load resistance *R* is:

(10) $$P=\frac{1}{RT}\int_{0}^{T}V^{2}\left(t\right)dt$$

in which, $T=\frac{D}{S_{t}U}$ is the time period of the separation-contact cycle.

### 4.3. Scaling Laws

Using the theoretical model for the TENG device, we may investigate the effect of each individual factor on the output performance of the device with the others fixed. However, the optimization work is restricted by the single-parameter analysis which does not reflect the relationship between the different parameters and the combined influence of them on the electrical output performance. In the simulation, the electric output performance is influenced by the typical factors (the dynamic characteristics of the pipe and spring-mass base for the device, the loading resistance in the electrical circuit and the material properties of the dielectric films in tribo-pair, etc.) simultaneously and coherently. The change of any factor may affect the optimized electric output performance. To overcome the limitation of single-parameter analysis and provide a general optimization method, we propose a new theoretical model to predict the output voltage and power of TENG in dimensionless forms. In this way, the output performance of TENG will be studied with all typical factors taken into consideration simultaneously.

To investigate the simultaneous effects of the typical parameters on output performance, here we establish a series of dimensionless expressions of the electric output of TENG device. In this theoretical model, all the typical factors are combined into dimensionless variables, including the gap distance of the tribo-pair. Based on Equation (8), the dimensionless expression for gap distance may be expressed as:

(11) $${\bar{y}}_{T1}(\tau )=\frac{m_{p}\kappa^{4}y_{T}(\tau )}{C_{L}\rho_{w}DU^{2}}=\frac{\gamma_{T}^{2}}{1-\gamma_{T}^{2}}\left(1+\sin2\pi t_{0}\tau \right)\sum_{j=1}^{n}\frac{\left(1-{\left(-1\right)}^{j}\right)\sin(j\pi \xi_{T})}{j^{5}\pi^{5}\sqrt{{\left(1-\gamma_{pj}{}^{2}\right)}^{2}+{\left(2\zeta_{pj}\gamma_{pj}\right)}^{2}}}$$

in which, the ratio of vortex shedding frequency to the natural frequency of pipe is $\gamma_{pj}$, while the ratio of vortex shedding frequency to the natural frequency of TENG is $\gamma_{T}$.

In Equation (11), ${\bar{y}}_{T1}(\tau )$ is the dimensionless expressions of the gap distance of the tribo-pair which is influenced by the installation position of TENG device at pipe $\xi_{T}$, the characteristics of ocean flow excitation ($C_{L}$, U, $\rho_{w}$), the pipe structure ($m_{p}$, D, $\kappa^{4}$), and frequency ratio ($\gamma_{T}$, $\gamma_{pj}$). Substituting Equation (10) into Equation (8), the dimensionless output voltage is:

(12) $$\begin{array}{l} \frac{V\left(\tau \right)\varepsilon_{0}m_{p}\kappa^{4}}{\sigma C_{L}\rho_{w}DU^{2}}={\bar{V}}_{B}\left(\tau ,\frac{m_{p}\kappa^{4}d_{0}}{C_{L}\rho_{w}DU^{2}},\frac{d_{0}t_{0}}{RS\varepsilon_{0}}\right)\\ \;=-\frac{m_{p}\kappa^{4}d_{0}}{C_{L}\rho_{w}DU^{2}}+\left(\frac{m_{p}\kappa^{4}d_{0}}{C_{L}\rho_{w}DU^{2}}+{\bar{y}}_{T1}(\tau )\right)\exp\left[-\frac{d_{0}t_{0}}{RS\varepsilon_{0}}\left(\tau +\frac{C_{L}\rho_{w}DU^{2}}{m_{p}\kappa^{4}d_{0}}\int_{0}^{\tau }{\bar{y}}_{T1}(\lambda )d\lambda \right)\right]\\ \;+\frac{d_{0}t_{0}}{RS\varepsilon_{0}}\left(\frac{m_{p}\kappa^{4}d_{0}}{C_{L}\rho_{w}DU^{2}}+{\bar{y}}_{T1}(\tau )\right)\int_{0}^{\tau }\exp\left[\frac{d_{0}t_{0}}{RS\varepsilon_{0}}\left(\left(\mu -\tau \right)+\frac{C_{L}\rho_{w}DU^{2}}{m_{p}\kappa^{4}d_{0}}\int_{\tau }^{\mu }{\bar{y}}_{T1}(\lambda )d\lambda \right)\right]d\mu \end{array}$$

Based on the normalized voltage, the dimensionless output power is given as follows:

(13) $$\frac{P\varepsilon_{0}m_{p}^{2}\kappa^{8}d_{0}t_{0}}{S\sigma^{2}C_{L}^{2}\rho_{w}^{2}D^{2}U^{4}}=P_{A}\left(\frac{m_{p}\kappa^{4}d_{0}}{C_{L}\rho_{w}DU^{2}},\frac{d_{0}t_{0}}{RS\varepsilon_{0}}\right)=\int_{0}^{1}{\bar{V}}_{A}^{2}d\tau$$

Equations (12) and (13) contain two compound variables, $\frac{m_{p}\kappa^{4}d_{0}}{C_{L}\rho_{w}DU^{2}}$ and $\frac{d_{0}t_{0}}{RS\varepsilon_{0}}$. The former mainly reflects the coherent influence of the dynamical characteristics of ocean flow excitation ($C_{L}$, U, $\rho_{w}$) and pipeline host (D, $\kappa^{4}$, $m_{p}$) on the dimensionless voltage, while the latter reflects influence of the structural properties of TENG (S, $\varepsilon_{0}$, $d_{0}$) and external circuit (*R*) on the output. Other corresponding parameters ($\xi_{T}$, $\gamma_{T}$, $\gamma_{pj}$) are not reflected directly in the two equations but they affect the output performance of TENG by influencing the gap distance of the tribo-pair according to Equation (10). The influence of $\xi_{T}$, $\gamma_{T}$, $\gamma_{pj}$ on the electric output performance is investigated using theoretical simulation in Section 5.

The relations in Equations (11)–(13) reveal the coherent influence of several mechanical and electrical factors on the electric output performance of TENG. However, there is still a limitation in the dimensionless expressions because the optimization of the structural characteristics of TENG device is not reflected in these equations. The mass $m_{T}$ of the base and the springs’ stiffness *k* are important factors to reflect the dynamic characteristics of TENG device, such as the natural frequency of the base which greatly influences the variation of the gap distance between the tribo-pair, thereby making a remarkable contribution to the electric output of TENG. To reveal the relationship among the electric output performance, the gap distance and the natural frequency of the base, we propose another expression for normalized gap distance with $m_{T}$ and *k* taken into consideration:

(14) $$\begin{array}{lll} {\bar{y}}_{T2}(\tau ) & = & \frac{m_{p}S_{t}^{2}y_{T}(\tau )}{C_{L}\rho_{w}D^{3}}\\ & = & \frac{4\pi^{2}}{\frac{t_{0}^{2}k}{m_{T}}-4\pi^{2}}\left(1+\sin\left(2\pi t_{0}\tau \right)\right)\sum_{j=1}^{n}\frac{\left(1-{\left(-1\right)}^{j}\right)\sin(j\pi \xi_{T})}{j^{5}\pi^{5}\sqrt{{\left(t_{0}^{2}\kappa^{4}-\frac{4}{j^{4}\pi^{2}}\right)}^{2}+{\left(2\zeta_{pj}\right)}^{2}\frac{4t_{0}^{2}\kappa^{4}}{j^{4}\pi^{2}}}} \end{array}$$

here, the dimensionless time $\tau =\frac{t}{t_{0}}$ and dimensionless installation position of the device at pipeline $\xi_{T}=\frac{x_{T}}{L}$ are defined, $\kappa^{4}=\frac{EI}{m_{p}L^{4}}$ is the square of the natural frequency of pipe, $t_{0}=\frac{D}{S_{t}U}$ the period of the current excitation.

As shown in Equation (14), ${\bar{y}}_{T2}(\tau )$ is influenced by the characteristics of ocean flow excitation ($C_{L}$, $S_{t}$, $\rho_{w}$), the pipe structure ($m_{p}$, D) and the TENG structure ($m_{T}$, *k*). Based on the dimensionless gap distance ${\bar{y}}_{T2}(\tau )$, the dimensionless expressions for voltage and power will be:

(15) $$\begin{array}{l} \frac{V\left(t\right)\varepsilon_{0}m_{p}S_{t}^{2}}{\sigma C_{L}\rho_{w}D^{3}}={\bar{V}}_{A}\left(\tau ,\frac{d_{0}m_{p}S_{t}^{2}}{C_{L}\rho_{w}D^{3}},\frac{d_{0}t_{0}}{RS\varepsilon_{0}}\right)\\ \;=-\frac{d_{0}m_{p}S_{t}^{2}}{C_{L}\rho_{w}D^{3}}+\left(\frac{d_{0}m_{p}S_{t}^{2}}{C_{L}\rho_{w}D^{3}}+{\bar{y}}_{T2}(\tau )\right)\exp\left[-\frac{d_{0}t_{0}}{RS\varepsilon_{0}}\left(\tau +\frac{C_{L}\rho_{w}D^{3}}{m_{p}S_{t}^{2}d_{0}}\int_{0}^{\tau }{\bar{y}}_{T2}(\lambda )d\lambda \right)\right]\\ \;+\frac{d_{0}t_{0}}{RS\varepsilon_{0}}\left(\frac{d_{0}m_{p}S_{t}^{2}}{C_{L}\rho_{w}D^{3}}+{\bar{y}}_{T2}(\tau )\right)\int_{0}^{\tau }\exp\left[\frac{d_{0}t_{0}}{RS\varepsilon_{0}}\left(\left(\mu -\tau \right)+\frac{C_{L}\rho_{w}D^{3}}{m_{p}S_{t}^{2}d_{0}}\int_{\tau }^{\mu }{\bar{y}}_{T2}(\lambda )d\lambda \right)\right]d\mu \end{array}$$

(16) $$\frac{P\varepsilon_{0}m_{p}^{2}S_{t}^{4}d_{0}t_{0}}{\sigma^{2}C_{L}^{2}\rho_{w}^{2}D^{6}S}=P_{A}\left(\frac{d_{0}m_{p}S_{t}^{2}}{C_{L}\rho_{w}D^{3}},\frac{d_{0}t_{0}}{RS\varepsilon_{0}}\right)=\int_{0}^{1}{\bar{V}}_{A}^{2}d\tau$$

According to Equations (14) and (15), we may understand the simultaneous effects of the loading resistance *R*, the area size of the dielectric materials *S*, the charge density σ, the effective thickness of the dielectric material films $d_{0}$, the characteristics of ocean flow excitation ($C_{L}$, $S_{t}$, $\rho_{w}$) and the pipe structure ($m_{p}$, D) on the dimensionless output voltage and power. We can also optimize the structural characteristics of TENG ($m_{T}$, *k*) to improve the electric output performance of TENG device through theoretical case study according to Equations (14)–(16).

In this section, we established two sets of dimensionless expressions of output performances with two dimensionless compound parameters to optimize the factors of the energy harvesting system. With Equations (11)–(16), we provide a general guideline for the optimization of the output performance of TENG, which may help us to understand the simultaneous influence of all typical factors on the electric output.

## 5. Theoretical Case Study

### 5.1. Validation of the Theoretical Model

**Figure 8:**
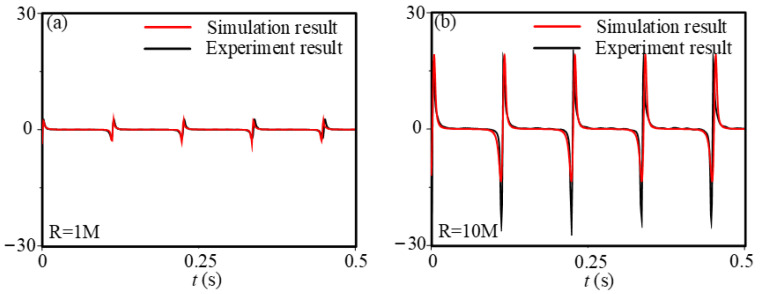
Comparison of the simulated and experimental voltage output with the loading resistance of (**a**) 1 MΩ and (**b**) 10 MΩ.

In Section 3, we tested the output performance of energy harvesting system with TENG devices, in which we obtain the electric output performance of TENG due to different mechanical or electrical conditions. As Figure 8 shows, the theoretical simulation for the output voltage of TENG is in high agreement with the experiments at either a loading resistance of 1 MΩ or 10 MΩ, which proved the accuracy of theoretical prediction method. Corresponding parameters are shown in Table 1 (see Section 3) and Table 2.

### 5.2. Individual Parameter Analysis of Output Performance

Further, we investigated the output performance of the energy harvesting system due to ocean current through theoretical analysis. The influence of four typical factors including the installation position of the device, the dynamic characteristics of the spring-mass base for the device, the loading resistance in the electrical circuit and the material properties of the dielectric films in tribo-pair on the electric output of TENG device are studied, which may provide guidelines for the design and arrangement of TENG devices on marine pipelines. The corresponding parameters are shown in Table 1 (see Section 3) and Table 2 (see Section 5.1).

**Figure 9:**
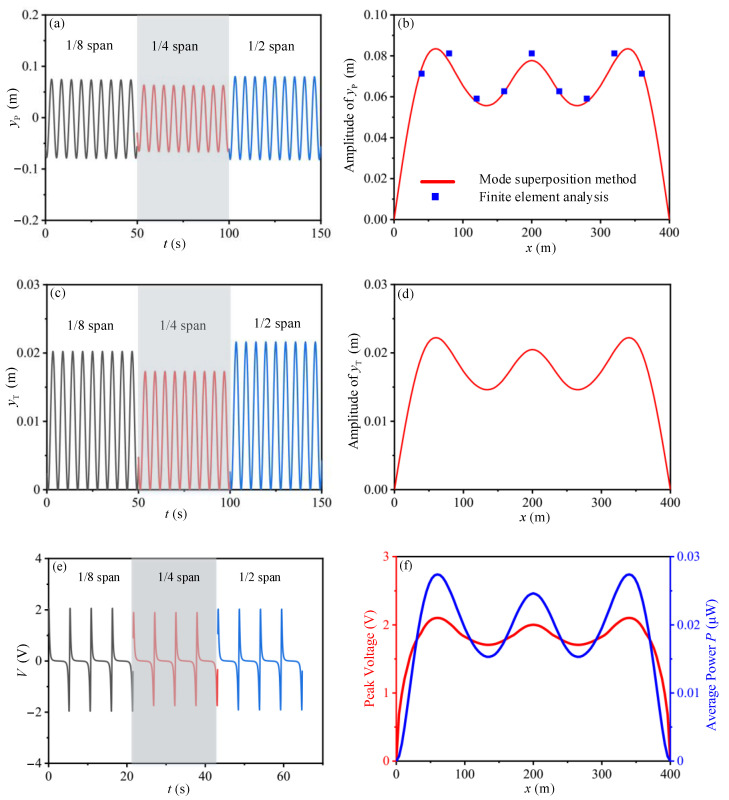
Influence of the installation position of the TENG device at pipeline on its output performance. (**a**,**b**) is the influence of the position at pipeline on the dynamic response of pipeline; (**c**,**d**) is the influence of the installation position of TENG at pipeline on the dynamic response of the mass-spring base; (**e**,**f**) is the influence of the installation position of TENG at pipeline on its electric output.

To obtain the feasible installation position of TENG devices on pipelines, the TENG devices are supposed to be arranged every two meters on a pipeline with the circuit resistance of 10 MΩ. The peak values for the dynamic response amplitude of pipeline, the oscillation response of the mass-spring base and the electric output of the TENG devices are shown in Figure 9, in which the time history analysis at 1/8, 1/4 and 1/2 span of the pipeline are presented, respectively.

Results show that the displacement amplitude of the pipeline, the gap distance between the tribo-pair, the peak voltage and average power of the TENG devices have similar trends as the installation positions are changed. The peak output voltage (2.1 V) and average output power (0.028 μW) of the TENG device reach their maximum values when *x* = 60 m and 340 m. When installed in the range of 40 m to 360 m, the TENG devices maintain a relatively high and stable electric output. That is to say, within this area, large quantities of TENG devices could be arranged to form a network to greatly improve the total electric output.

**Figure 10:**
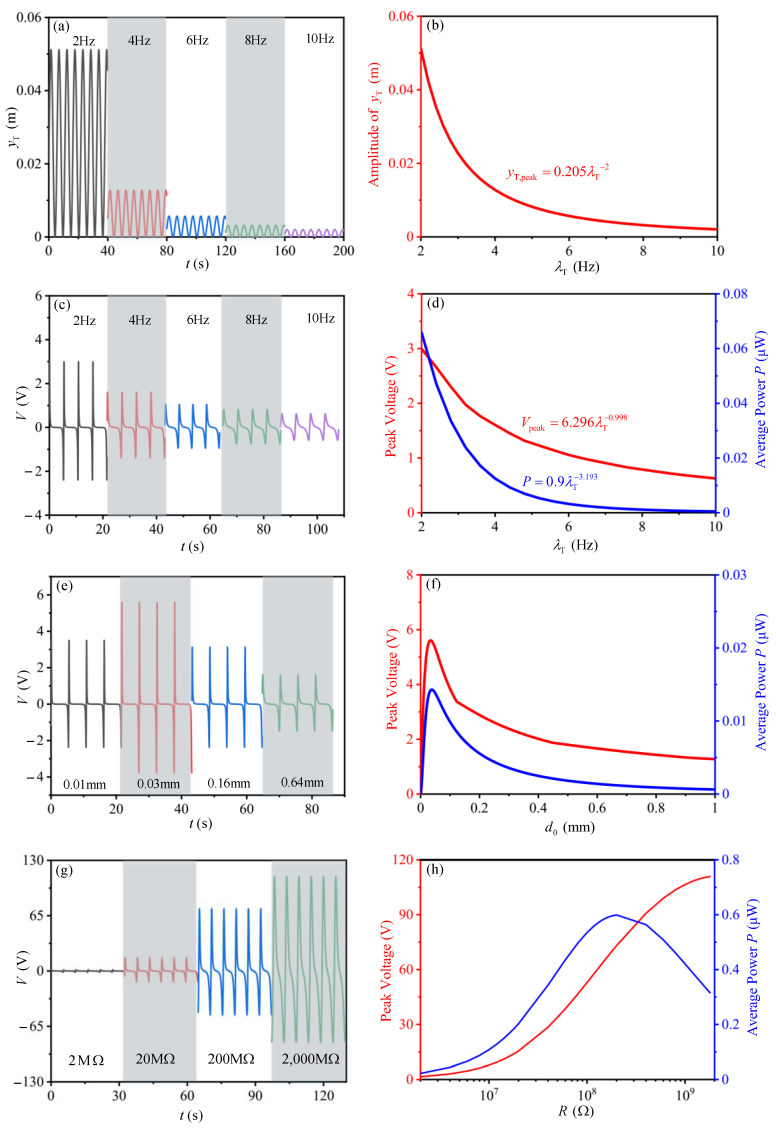
Influence of the natural frequency of the mass-spring bass, effective thickness of the dielectric films and the circuit resistance on the output performance of TENG device. (**a**,**b**) are the influence of the natural frequency of the mass-spring base on the dynamic response of the base; (**c**,**d**) are the influence of the natural frequency of the mass-spring base on the electric output of TENG device; (**e**,**f**) are the influence of the effective thickness of the dielectric layer on the electric output of TENG device; (**g**,**h**) are the influence of the circuit loading resistance on the electric output of TENG device.

As one of the factors which may greatly affect the electric output of TENG device, the time histories of the gap distance between the tribo-pair is dependent on the dynamic characteristics of the mass-spring base. To provide an accordance for the structural design of the mass-spring base, we investigate the output performance of TENG device with different natural frequencies (Figure 10a–d). As shown in Figure 10b, with increasing of the natural frequency, the amplitude for the gap distance between the tribo-pair shows exponential drop, which could be expressed by the equation of $y_{T,\max}=0.205\lambda_{T}^{-2}$. The peak voltage and average power also show exponential drop with the expression of $V_{\mathrm{peak}}=6.296\lambda_{T}^{-0.998}$ and $P=0.9\lambda_{T}^{-3.193}$, respectively (Figure 10d). The results indicate that reducing the natural frequency of the mass-spring base may help improve the output performance of the TENG device.

The influence of the effective thickness of the dielectric layer *d*_0_ is also studied (Figure 10f), the peak voltage and average power have similar variation trends with the increase of *d*_0_. The peak voltage (5.6 V) and power (0.014 μW) reaches its maximum value when *d*_0_ is 0.036 mm. The corresponding results provide an accordance for the design of dielectric films in tribo-pair. In circuit design analysis, the best electrical resistance for the system is 180 MΩ (Figure 10h).

### 5.3. Multiple Parameters Analysis of Output Performance

Based on the dimensionless expressions of the electrical output, the theoretical analysis is developed to reveal a clear correlation between the normalized output performance and the dimensionless compound parameters using Equations (11)–(16). The simulation is carried out using multi-parameter analysis, so that it can be utilized in the design for the energy harvesting system with any structure or material parameters.

**Figure 11:**
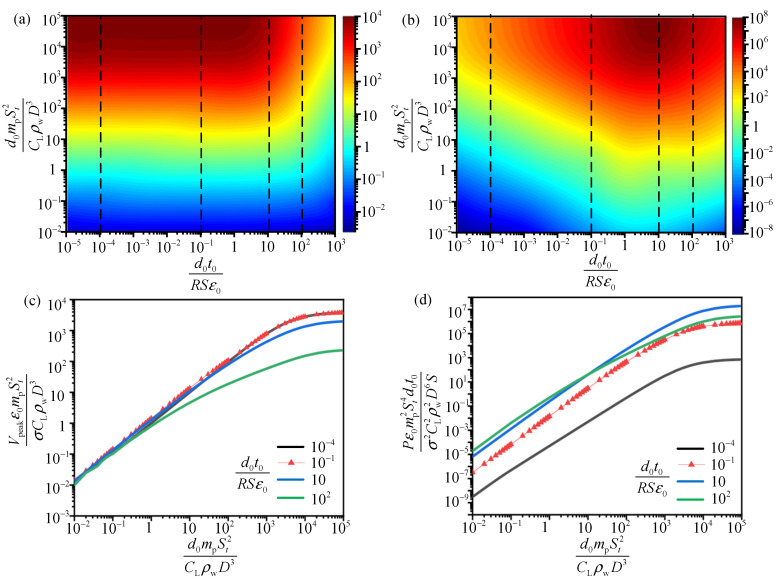
Analysis of the output performance ideal energy harvesting system with dimensionless parameters. (**a**,**b**) are power maps for the two dimensionless parameters $\frac{d_{0}t_{0}}{RS\varepsilon_{0}}$ and $\frac{d_{0}m_{p}S_{t}^{2}}{C_{L}\rho_{w}D^{3}}$. The color represents the dimensionless peak voltage ${\bar{V}}_{n1}$ and average power ${\bar{P}}_{n1}$, respectively. (**c**,**d**) show restricted optimization voltage and power versus $\frac{d_{0}m_{p}S_{t}^{2}}{C_{L}\rho_{w}D^{3}}$ with $\frac{d_{0}t_{0}}{RS\varepsilon_{0}}$ fixed.

The scaling laws relating the dimensionless output and the two compound variables ($\frac{d_{0}t_{0}}{RS\varepsilon_{0}}$ and $\frac{d_{0}m_{p}S_{t}^{2}}{C_{L}\rho_{w}D^{3}}$) are presented in Figure 11. According to the possible range of the corresponding parameters of TENG and the pipe in actual applications, the compound parameter $\frac{d_{0}t_{0}}{RS\varepsilon_{0}}$ is evaluated in the range of $10^{-5}$ to $10^{2}$ with $\frac{d_{0}m_{p}S_{t}^{2}}{C_{L}\rho_{w}D^{3}}$ in the range of $10^{-2}$ to $10^{5}$.

In Figure 11a,b for the relationship between the compound variables ($\frac{d_{0}m_{p}S_{t}^{2}}{C_{L}\rho_{w}D^{3}}$ and $\frac{d_{0}t_{0}}{RS\varepsilon_{0}}$) on the dimensionless output, it is obvious that the increasing of $\frac{d_{0}m_{p}S_{t}^{2}}{C_{L}\rho_{w}D^{3}}$ may enhance the dimensionless peak voltage ${\bar{V}}_{n1}$ ($\frac{V_{\mathrm{peak}}\varepsilon_{0}m_{p}S_{t}^{2}}{\sigma C_{L}\rho_{w}D^{3}}$) and power ${\bar{P}}_{n1}$ ($\frac{P\varepsilon_{0}m_{p}^{2}S_{t}^{4}d_{0}t_{0}}{\sigma^{2}C_{L}^{2}\rho_{w}^{2}D^{6}S}$) no matter how $\frac{d_{0}t_{0}}{RS\varepsilon_{0}}$ varies. When $\frac{d_{0}m_{p}S_{t}^{2}}{C_{L}\rho_{w}D^{3}}$ is in the range of $10^{4}\text{–}10^{5}$ and $\frac{d_{0}t_{0}}{RS\varepsilon_{0}}$ in the range of 1–10, both the dimensionless voltage and power reaches the optimal value. Thus, as long as the two compound variables are evaluated in these ranges through device design and parameter analysis, the electric output of TENG may be maximized.

For better understanding of the influence of $\frac{d_{0}m_{p}S_{t}^{2}}{C_{L}\rho_{w}D^{3}}$ on electric output performance of TENG, we present the restricted optimization voltage and power versus $\frac{d_{0}m_{p}S_{t}^{2}}{C_{L}\rho_{w}D^{3}}$ with $\frac{d_{0}t_{0}}{RS\varepsilon_{0}}$ fixed (Figure 11c,d). As can be seen, for each $\frac{d_{0}t_{0}}{RS\varepsilon_{0}}$, both the dimensionless voltage ${\bar{V}}_{n1}$ and power ${\bar{P}}_{n1}$ increase rapidly with $\frac{d_{0}m_{p}S_{t}^{2}}{C_{L}\rho_{w}D^{3}}$ firstly, and then tend to a stable value when $\frac{d_{0}m_{p}S_{t}^{2}}{C_{L}\rho_{w}D^{3}}$ reaches $10^{4}$. Thus, the optimal range of $\frac{d_{0}m_{p}S_{t}^{2}}{C_{L}\rho_{w}D^{3}}$ is $10^{4}–10^{5}$. In real applications, the Strouhal number $S_{t}$, water density $\rho_{w}$ and Lift coefficient $C_{L}$ in this variable reflect the characteristics of the ocean flow and may be not adjustable. For optimization of the output performance, we can design the structural characteristics of the pipe ($m_{p}$, D) and TENG ($d_{0}$) to make the compound variable in the optimal range.

**Figure 12:**
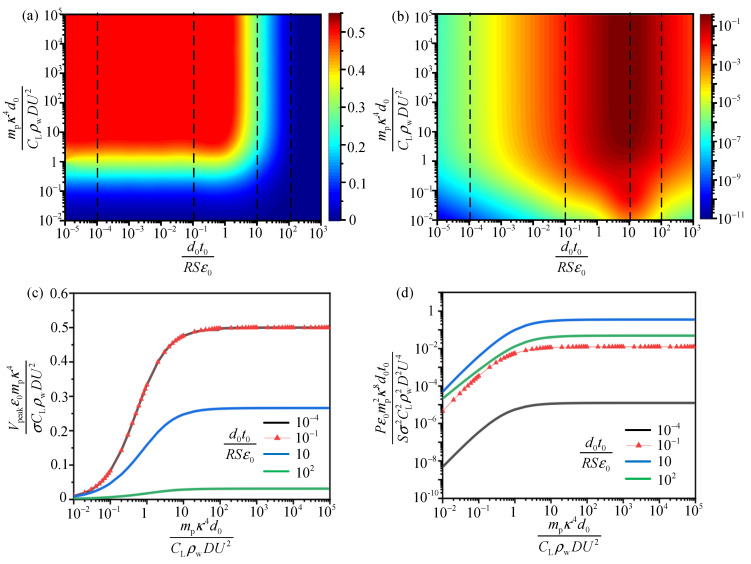
Analysis of the output performance ideal energy harvesting system with dimensionless parameters. (**a**,**b**) are power maps for the two dimensionless parameters $\frac{d_{0}t_{0}}{RS\varepsilon_{0}}$ and $\frac{m_{p}\kappa^{4}d_{0}}{C_{L}\rho_{w}DU^{2}}$. The color represents the dimensionless peak voltage ${\bar{V}}_{n2}$ and average power ${\bar{P}}_{n2}$, respectively; (**c**,**d**) show restricted optimization voltage and power versus $\frac{m_{p}\kappa^{4}d_{0}}{C_{L}\rho_{w}DU^{2}}$ with $\frac{d_{0}t_{0}}{RS\varepsilon_{0}}$ fixed.

To investigate the relationship between the output performance and the dynamic characteristics of the system in detail, another scaling law is exhibited in Figure 12 based on Equations (11)–(13). The results show that the dimensionless compound parameters ($\frac{m_{p}\kappa^{4}d_{0}}{C_{L}\rho_{w}DU^{2}}$ and $\frac{d_{0}t_{0}}{RS\varepsilon_{0}}$) have a larger optimal range for the best output performance than that in Figure 11. The dimensionless voltage ${\bar{V}}_{n2}$ ($\frac{V_{\mathrm{peak}}\varepsilon_{0}m_{p}\kappa^{4}}{\sigma C_{L}\rho_{w}DU^{2}}$) in Figure 12a reaches to its maximum value (0.5) when $\frac{m_{p}\kappa^{4}d_{0}}{C_{L}\rho_{w}DU^{2}}$ ranges in $1\text{–}10^{5}$ with $\frac{d_{0}t_{0}}{RS\varepsilon_{0}}$ from $10^{-5}$ to 4. The dimensionless power reaches the maximum value when $\frac{m_{p}\kappa^{4}d_{0}}{C_{L}\rho_{w}DU^{2}}$ ranges from 10 to $10^{5}$ with $\frac{d_{0}t_{0}}{RS\varepsilon_{0}}$ ranging from 1 to $10^{2}$. In Figure 12c,d, for each $\frac{d_{0}t_{0}}{RS\varepsilon_{0}}$, the value of ${\bar{V}}_{n2}$ and ${\bar{P}}_{n2}$ ($\frac{P\varepsilon_{0}m_{p}^{2}\kappa^{8}d_{0}t_{0}}{S\sigma^{2}C_{L}^{2}\rho_{w}^{2}D^{2}U^{4}}$) increases monotonically with $\frac{m_{p}\kappa^{4}d_{0}}{C_{L}\rho_{w}DU^{2}}$ and approaches to a constant when $\frac{m_{p}\kappa^{4}d_{0}}{C_{L}\rho_{w}DU^{2}}=10$, which implies that optimal $\frac{m_{p}\kappa^{4}d_{0}}{C_{L}\rho_{w}DU^{2}}$ is over 10. In short, when $\frac{m_{p}\kappa^{4}d_{0}}{C_{L}\rho_{w}DU^{2}}$ ranges in $10\text{–}10^{5}$ with $\frac{d_{0}t_{0}}{RS\varepsilon_{0}}$ from 1 to 4, both the dimensionless power and voltage reaches the maximum value simultaneously. Arranging the two variables in these ranges, we may obtain the best output voltage and power of TENG device at the same time.

Based on Figure 11 and Figure 12, we obtain the optimal range of $\frac{d_{0}t_{0}}{RS\varepsilon_{0}}$, $\frac{d_{0}m_{p}S_{t}^{2}}{C_{L}\rho_{w}D^{3}}$ and $\frac{m_{p}\kappa^{4}d_{0}}{C_{L}\rho_{w}DU^{2}}$ for the best dimensionless voltage and power of TENG device, respectively. Besides, we can also carry out single-parameter analysis to improve the output performance based on the results. For example, the influence of current excitation period $t_{0}$, area *S* of the tribo-pair, load resistance *R* and the effective thickness of the dielectric material films $d_{0}$ on the output voltage ${\bar{V}}_{n1}$ can be realized in Figure 11a,b. After obtaining the best combination of $\frac{d_{0}t_{0}}{RS\varepsilon_{0}}$ and $\frac{d_{0}m_{p}S_{t}^{2}}{C_{L}\rho_{w}D^{3}}$, we can study one of above parameters ($t_{0}$, *S*, *R*, *D*, $m_{p}$ and $d_{0}$) with other parameters remaining fixed.

**Figure 13:**
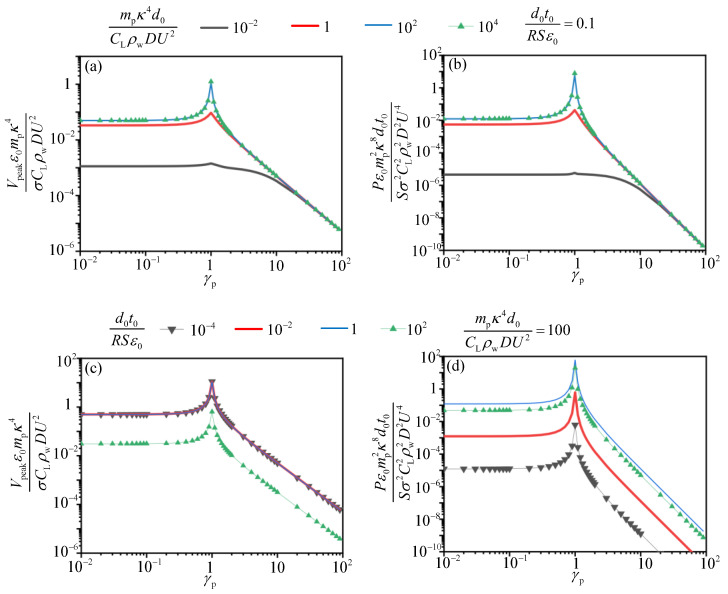
The normalized voltage ${\bar{V}}_{n2}$ and ${\bar{P}}_{n2}$ versus $\gamma_{p}$. (**a**,**b**) are normalized voltage and power versus $\gamma_{p}$ with $\frac{d_{0}t_{0}}{RS\varepsilon_{0}}$ fixed; (**c**,**d**) are normalized voltage and power versus $\gamma_{p}$ with $\frac{m_{p}\kappa^{4}d_{0}}{C_{L}\rho_{w}DU^{2}}$ fixed.

**Figure 14:**
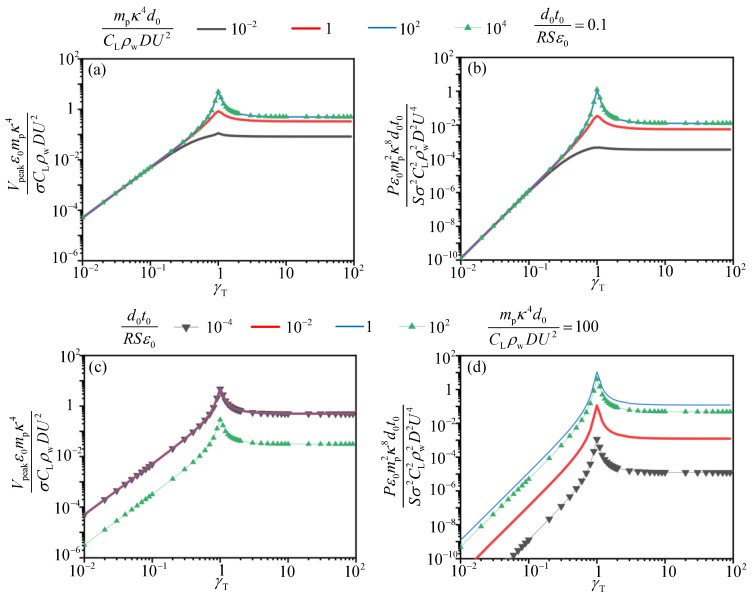
The normalized voltage ${\bar{V}}_{n2}$ and ${\bar{P}}_{n2}$ versus $\gamma_{T}$. (**a**,**b**) are normalized voltage and power versus $\gamma_{T}$ with $\frac{d_{0}t_{0}}{RS\varepsilon_{0}}$ fixed; (**c**,**d**) are normalized voltage and power versus $\gamma_{T}$ with $\frac{m_{p}\kappa^{4}d_{0}}{C_{L}\rho_{w}DU^{2}}$ fixed.

To investigate the influence of frequency ratio between the flow excitation, the pipe and TENG on the output performance, the normalized voltage and power versus $\gamma_{p}$ and $\gamma_{T}$ with specific $\frac{d_{0}t_{0}}{RS\varepsilon_{0}}$ and $\frac{m_{p}\kappa^{4}d_{0}}{C_{L}\rho_{w}DU^{2}}$ are presented in Figure 13 and Figure 14. $\frac{d_{0}t_{0}}{RS\varepsilon_{0}}$ and $\frac{m_{p}\kappa^{4}d_{0}}{C_{L}\rho_{w}DU^{2}}$ are selected in the range where normalized voltage and power reaches the optimal value according to Figure 12a,b. It is observed that all voltage and power curves reach the maximum values when $\gamma_{p}=1$, which will lead to the resonance of pipe and the device. The similar tendency of the dimensionless voltage and power versus the frequency ratio between the current excitation and the mass-spring base of TENG device is shown in Figure 14. In practice, the resonance phenomenon is harmful for the structural safety of the system and should be prevented. However, when $\gamma_{p}$ is less than 1 and $\gamma_{T}$ greater than 1 the dimensionless voltage and power both reach a considerable and stable value. We can carry out structural design to make the energy harvesting system achieve this value, thereby enhancing the electric output of the TENG device.

## 6. Conclusions

In this paper, we use a contact-mode triboelectric nanogenerator (TENG) to scavenge mechanical energy from vibrating marine pipes. The TENG device is fabricated with PTFE, nylon dielectric material films, acrylic base, springs and a waterproof box. The output performance of the TENG is tested by experiment means, which proves the feasibility of TENG. Theoretical simulations are carried out to investigate the influence of typical factors on the electric output, such as the dynamic characteristics of the pipe and spring-mass base for the device, the loading resistance in the electrical circuit and the material properties of the dielectric films in tribo-pair. In addition, two groups of dimensionless expressions for normalized electric output versus two compound variables are established to provide a general optimization method of the energy harvesting system. Based on the experiment tests and theoretical simulation in this paper, the main conclusion is obtained as below:

(1) The experimental tests confirm that the electrical output of TENG may meet the requirement of the monitoring system in pipes. The output power of TENG reaches the maximum value of 14.0 μW with the power density of 5.56 mW/m^2^ at the electrical circuit resistance of 200 MΩ. If all TENG devices are connected in series with large quantities to form a network, the output of the energy harvesting system will be enhanced greatly to provide adequate energy for the monitoring system in pipes.

(2) The output performance of TENG is further investigated by theoretical simulations, with the influence of four typical factors on the output performance of TENG devices obtained. It is observed that the peak dynamic response amplitude of occurs near the 1/8 span of pipe. Thus, installing TENG devices near the 1/8 span of the pipe may help maximize the electric output. The results also provide a guideline for structural and material design for TENG devices. Reducing the natural frequency of the mass-spring base can help enhance the electric output of TENG devices, and the optimal effective thickness of the tribo-pair is 0.036 mm. Moreover, it is found that the maximum output power appears at the resistance of 180 MΩ, which is close to the resistance (200 MΩ) in the experiment and proves again the accuracy of the theoretical model.

(3) The multiple-parameters analysis reveals the relationship between the normalized electric output and the normalized system parameters. With regard to first set of the scaling law, both the normalized voltage ${\bar{V}}_{n1}$ and power ${\bar{P}}_{n1}$ reaches their maximum value simultaneously when the compound variable $\frac{d_{0}m_{p}S_{t}^{2}}{C_{L}\rho_{w}D^{3}}$ ranges from $10^{4}$ to $10^{5}$ with $\frac{d_{0}t_{0}}{RS\varepsilon_{0}}$ in the range of 1–10. For another set of dimensionless expressions, the normalized voltage ${\bar{V}}_{n2}$ and power ${\bar{P}}_{n2}$ reaches the maximum value with the compound variable $\frac{m_{p}\kappa^{4}d_{0}}{C_{L}\rho_{w}DU^{2}}$ in the range of $10\text{–}10^{5}$ and $\frac{d_{0}t_{0}}{RS\varepsilon_{0}}$ in the range of 1 to 4. Moreover, the influence of the natural frequency of the system on the electric output of TENG is investigated. It is observed that TENG has a considerable and stable electric output when $\gamma_{p}$ is less than 1 or $\gamma_{T}$ greater than 1. Arranging these variables in their optimal ranges through structural design and parameters adjustment, the best normalized voltage and power may be achieved.

The feasibility of using TENG to scavenge mechanical energy from marine pipes is proved by experiment tests and theoretical simulations. Through the theoretical simulation, the optimal ranges for the typical factors are presented, which provides a guideline for the structural and material design of the proposed energy harvesting system. In the future, the application of TENG devices is expected to be a feasible approach to power monitoring systems for marine pipelines.

## Figures and Tables

**Table 1:** Parameters of pipe and the energy harvesting system.

Parameter	Value
Pipe length *L*	1.2 m
Pipe diameter *D*	0.02 m
$\mathrm{Dielectric}\;\mathrm{constant}\;\mathrm{of}\;\mathrm{PTFE}\;\varepsilon_{r1}$	2.55
$\mathrm{Dielectric}\;\mathrm{constant}\;\mathrm{of}\;\mathrm{nylon}\;\varepsilon_{r2}$	4.55
$\mathrm{Thickness}\;\mathrm{of}\;\mathrm{PTFE}\;d_{1}$	0.05 mm
$\mathrm{Thickness}\;\mathrm{of}\;\mathrm{nylon}\;d_{2}$	0.05 mm
Number of springs n	4
$\mathrm{Number}\;\mathrm{of}\;\mathrm{spring}\;\mathrm{coils}\;N_{c}$	8
$\mathrm{Spring}\;\mathrm{diameter}\;D_{c}$	4 mm
$\mathrm{Spring}\;\mathrm{wire}\;\mathrm{diameter}\;d_{c}$	0.15 mm
$\mathrm{Mass}\;\mathrm{of}\;\mathrm{the}\;\mathrm{mass}-\mathrm{spring}\;\mathrm{base}\;m_{T}$	19.46 g
Natural frequency of the TENG f	7.1 Hz
Circuit resistance R	100 MΩ
$\mathrm{Vacuum}\;\mathrm{dielectric}\;\text{co-efficient}\;\varepsilon_{0}$	8.854 × 10^−12^
Surface charge density σ	5–20 μC/m^2^
Temperature *T*	20 °C
Humidity	40%
Dimensions of tribo-pair	5 cm × 5 cm

**Table 2:** Parameters of the marine pipeline and ocean current.

Parameter	Value
Pipe length	400 m
The external diameter of pipeline	0.324 m
The inner diameter of pipeline	0.284 m
The water current velocity	0.3 m/s
Lift coefficient of the current	0.9

## Data Availability

Not applicable.

## Supplementary Materials

The following are available online at https://www.mdpi.com/1424-8220/21/4/1514/s1, Video S1: LEDs powered by TENG.
